# The mutual history of Schlegel’s Japanese gecko (Reptilia: Squamata: Gekkonidae) and humans inscribed in genes and ancient literature

**DOI:** 10.1093/pnasnexus/pgac245

**Published:** 2022-11-30

**Authors:** Minoru Chiba, Takahiro Hirano, Daishi Yamazaki, Bin Ye, Shun Ito, Osamu Kagawa, Komei Endo, Shu Nishida, Seiji Hara, Kenichiro Aratake, Satoshi Chiba

**Affiliations:** Graduate School of Life Sciences, Tohoku University, Miyagi, Japan; Graduate School of Life Sciences, Tohoku University, Miyagi, Japan; Center for Northeast Asian Studies, Tohoku University, Miyagi, Japan; Center for Northeast Asian Studies, Tohoku University, Miyagi, Japan; Faculty of Agriculture and Marine Science, Kochi University, Kochi, Japan; Graduate School of Life Sciences, Tohoku University, Miyagi, Japan; Institute of Biomedical and Health Engineering, Shenzhen Institute of Advanced Technology, Chinese Academy of Sciences, Shenzhen, China; Graduate School of Life Sciences, Tohoku University, Miyagi, Japan; Center for Northeast Asian Studies, Tohoku University, Miyagi, Japan; Graduate School of Life Sciences, Tohoku University, Miyagi, Japan; Shimoda Marine Research Center, University of Tsukuba, Shizuoka, Japan; Graduate School of Life Sciences, Tohoku University, Miyagi, Japan; Graduate School of Life Sciences, Tohoku University, Miyagi, Japan; Graduate School of Life Sciences, Tohoku University, Miyagi, Japan; Center for Northeast Asian Studies, Tohoku University, Miyagi, Japan; Graduate School of Life Sciences, Tohoku University, Miyagi, Japan; Center for Northeast Asian Studies, Tohoku University, Miyagi, Japan

**Keywords:** ancient literature, ddRAD-seq, phylogeography, gecko, human-mediated dispersal

## Abstract

Knowing how the present distribution of organisms was formed is an essential issue in evolutionary ecology. Recently, the distribution of organisms on Earth has been significantly changed by human-mediated dispersal due to globalization. Therefore, significant attention has been paid to such processes. However, although humankind has taken considerable time to achieve modernization, the impact of ancient human activity on ecosystems has not yet been thoroughly studied. We hypothesized that ancient urban development and transitions had a non-negligible effect on species distribution. Inferring the impact of past human activity on ecosystems from ancient literature and verifying that impact by genetic analysis and human history is an effective means of tackling this problem. As geckos, a popular neighbor of human dwellings, are good material for this model, we performed this combination approach using Schlegel’s Japanese gecko, *Gekko japonicus*. We show that *G. japonicus* migrated from China to the western Japanese archipelago before Christ. The gecko species dispersed itself from western to eastern the archipelago on a time scale of thousands of years. There are many synchronizations between the dispersal history of *G. japonicus* and the historical development of human society. It is suggested by such synchronizations that humans have influenced the distribution of *G. japonicus* many times throughout its dispersal history.

Significance Statement How have humans affected biogeography over a long history? Not only biology, but also the humanities can help answer this question. Just as today, ancient people were interested in diverse organisms. By deciphering ancient literature, we can infer the relationships between humans and organisms at that time, but it is rare for natural scientists to pay attention to such sources. Here, we genetically and anthropologically verified hypotheses about biogeography designed from ancient literature. This combination analysis revealed that a gecko species has artificially expanded its distribution in Japan via different events throughout thousands of years. A paradigm shift is provided by this knowledge; ancient human–organism interactions can be an essential factor in understanding the present distribution of organisms.

## Introduction

How has the current distribution of organisms been affected by human activities? The answer to this question is essential to understanding the origins of biodiversity (1). Sometimes, species have been enabled by human-mediated dispersal to travel much faster and further than their natural dispersal ability (2, 3). There are countless organisms whose distributions are artificially expanding worldwide (4, 5). However, many of the cases that have received attention are recent events, and there are not many studies from the perspective of evolutionary biology on how human activity has affected organisms over the long history of humans (6–8).

Schlegel’s Japanese gecko, *Gekko japonicus* (Reptilia: Squamata: Gekkonidae) is widely distributed in eastern China, South Korea, and south of the Tohoku region within the Japanese archipelago. Recently, Japanese populations of this species have been considered to be derived from an old immigrant Chinese population. This hypothesis has been supported by morphological and molecular genetic studies (9, 10). It has been suggested by paleospecies distribution modeling that *G. japonicus* was able to settle in Japan after the middle Holocene when the climate became warmer (10). Due to the habitat preference of *G. japonicus* for artificial environments, such as buildings and cargo (11), its migration and expansion from China to Japan are predicted to have been affected by ancient human activities.

Here, we conducted a study to clarify the dispersal history of *G. japonicus* in the Japanese archipelago, thought to have been influenced by human activity over a long-term period. Recently, deciphering historical materials, such as ancient literature, has been emphasized in assessing the effects of humans on past biodiversity and ecosystems (12, 13). *Gekko japonicus*, a popular organism living close to humans, is variously recorded in ancient Japanese literature. Therefore, we first comprehensively deciphered ancient Japanese literature and collected descriptions of reptiles and amphibians (it seems that the two were indistinguishable to ancient peoples). Based on this ancient knowledge, we hypothesized that *G. japonicus* was introduced to western Japan by at least the 900s AD, and its distribution expanded to eastern Japan relatively recently, after the 1700s AD. Specimens were then collected from various parts of the Japanese archipelago and China, and a population genetic analysis was performed using genotyping by sequencing (GBS). The dispersal scenario estimated from population genetics was compared with the hypothesis based on ancient literature. Finally, the validity of our findings was verified from the viewpoint of human history. Through a combined approach of the humanities and biology, we have clarified the long-term effects of humans on the distribution of *G. japonicus*.

## Results

### Scrutiny of ancient literature

**Fig. 1.**
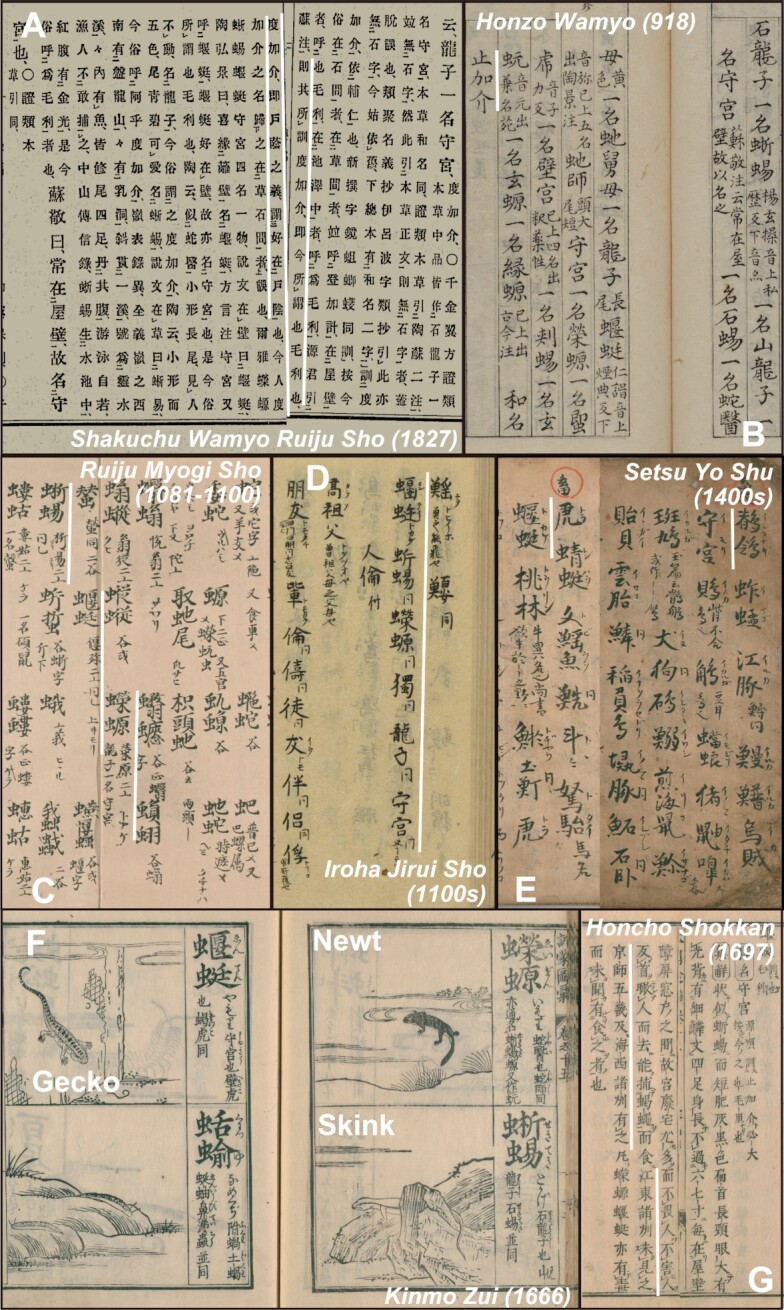
Ancient Japanese literature with a description of lizards. The highlighted descriptions (white) are outlined in the text. (A) *Shakuchu Wamyo Ruiju Sho*, a document with an annotated dictionary published in the 900s AD. (B) *Honzo Wamyo*, the oldest Japanese pharmacology dictionary. (C)–(E) *Ruiju Myogi Sho, Iroha Jirui Sho*, and *Setsu Yo Shu*, are Chinese–Japanese dictionaries from ancient to medieval times. (F) *Kinmo Zui*, an encyclopedia with pictures published in the early modern period. (G)*Honcho Shokkan*, a medical book published in the early modern period.

Descriptions of lizards, including geckos, have been found in dozens of ancient documents of various ages, and the most significant examples are shown in Fig. 1. As shown in the descriptions in Fig. 1(A), the Japanese name “tokage,” which now means “skink,” comes from the word for something that is behind a door and may originally have represented a gecko (14, 15). The word “tokage” had already appeared in the 10th century (Fig. 1B), suggesting that its existence has been known for a long time (16). We can see from the examples in Fig. 1(C)–(E) that two lizards (skinks and geckos) and one amphibian (newts) were often confused in Japan before 1600 AD, and the notations differed in each document (17–21). “Geckos” were called “yamori” around the 15th century (22). Since then, the above three have been distinguished, as shown in Fig. 1(F) (23, 24). On the other hand, it is suggested from Fig. 1(G) that geckos inhabit areas nearby Kyoto and Kyushu (western Japan), but have not been found in the Kanto region (eastern Japan) as of 1697 (25). This past distribution is shown in Fig. 4(C).

### De-novo assembly, phylogenetic analysis, and population structure

The average raw reads were Set.Phylogeny: 3,360,142, Set.Structure1: 3,357,559, Set.Structure2: 3,182,293, and Set.Divergence: 3,286,725. The respective total filtered loci of each dataset were 8464,8174, 19,462, and 8,592. The number of single nucleotide polymorphisms (SNPs) was 66,991 (2.82% missing sites), 29,828 (2.37% missing sites), 50,167 (4.15% missing sites), and 30,477 (2.43% missing sites), respectively.

**Fig. 2.**
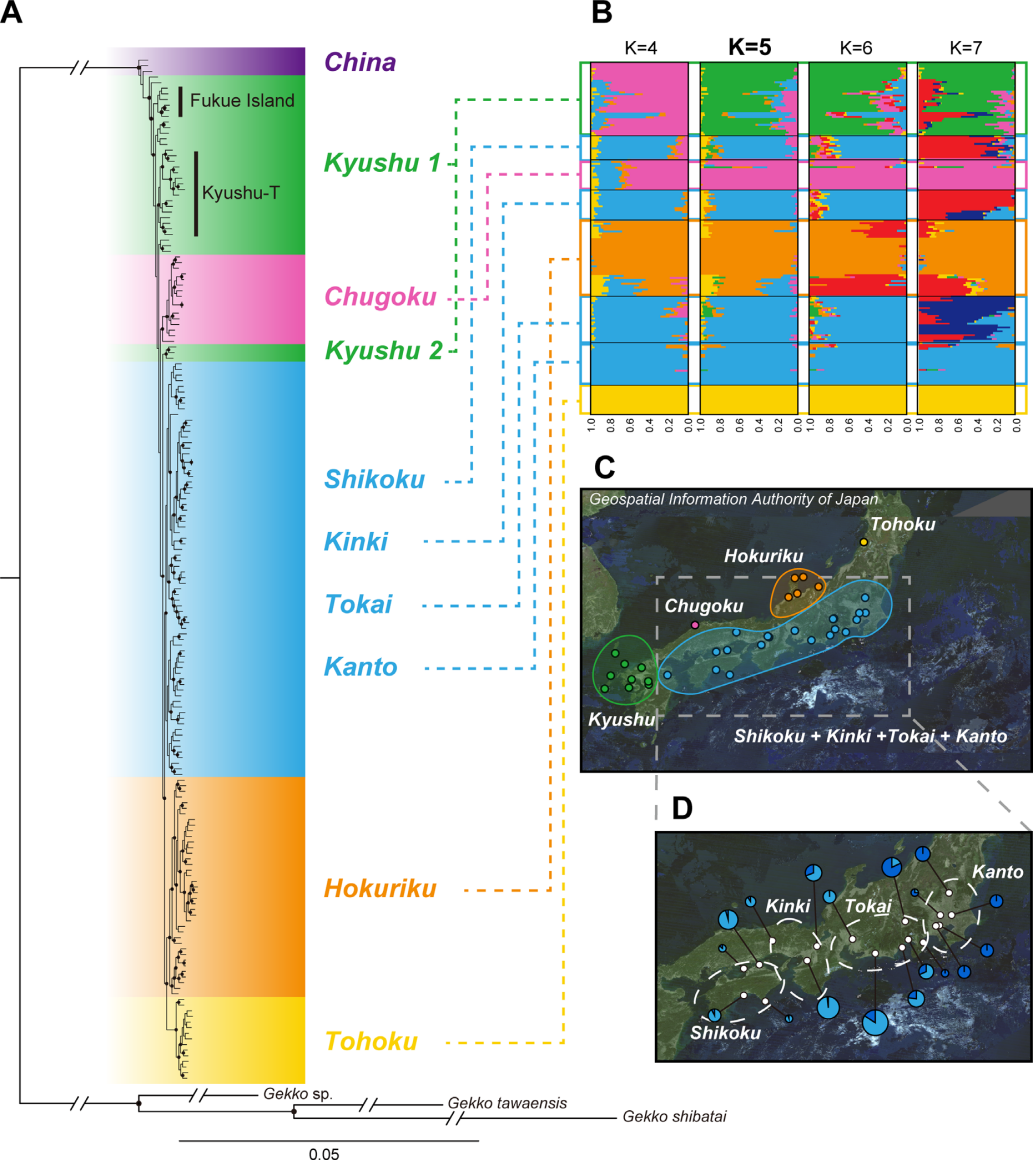
Phylogeographic information of *G. japonicus*. (A) A molecular phylogenetic tree based on the ML method and the approximate Bayesian method. Nodes with a closed circle represent Bayes posterior probabilities higher than 0.99 and UBPP higher than 95%. The Fukue Island and Kyushu-T clades are monophyletic groups that were considered separately in the divergence time and demographic history analyses. (B) Genetic structure of the Japanese populations of *G. japonicus*. Each bar on the horizontal axis represents an individual, and the vertical axis represents the probability that an individual belongs to each cluster (each color). The lowest observed cover error was K = 5. (C) Mapped geographical genetic structure of the Japanese population. (D) Geographical genetic structure of the Shikoku, Kinki, Tokai, and Kanto regions when K = 2. The breakdown of the pie chart represents the probability that an individual belongs to each cluster. The diameter of the pie chart represents the number of individuals at each site.

The phylogenetic tree, based on maximum likelihood and the approximate Bayesian method, is shown in Fig. 2(A). Through Model Finder, TVM + F + R10 was selected as the replacement model with the smallest Bayesian information criteria (BIC). In most cases, individuals collected from the same site formed a monophyletic group. Looking at the phylogenetic relationships, the individuals in Nanjing, China, were the most ancestral but did not form an independent monophyletic group. The Kyushu population, excluding Beppu, was the eldest ancestor population of the Japanese archipelago. This clade was regarded as Kyushu 1. Only the Beppu population was phylogenetically far away from Kyushu 1, and that was regarded as Kyushu 2. Still, individuals from Kyushu 1 did also not form a single monophyletic group. Also, while the populations of remote islands, such as Fukue Island and Tsushima, were geographically differentiated from island to island, they were not so differentiated on the mainland of Kyushu. Although we could collect from only one site (Izumo) in the Chugoku region, all the individuals collected from this area formed a monophyletic group. Individuals from Kinki, Kanto, Tokai, and Shikoku formed one large clade. Individuals from the Hokuriku and Tohoku regions formed separate monophyly groups and were geographically differentiated. The support for the nodes in each regional clade, which are color-coded in Fig. 2(A) was Bayes posterior probabilities higher than 0.99 and Ultrafast bootstrap posterior probability (UBPP) (26) higher than 95%.

The results of the ADMIXTURE (27) analysis of the Japanese archipelago are shown in Fig. 2(B). The lowest value of cover error was at K  =  5 (0.39297), and the next lowest values were at K = 4, 6, and 7 (0.39369, 0.39637, and 0.39403). Geographically differentiated genetic structures similar to those in the molecular phylogenetic analysis were found. However, at K = 5, the populations of the Shikoku, Kinki, Tokai, and Kanto regions were all grouped into one cluster. This geographical structure is shown in Fig. 2(C). As a result of a detailed ADMIXTURE analysis in the Shikoku, Kinki, Tokai, and Kanto regions, K = 1 had the slightest cover error (0.53364), and the next lowest values, as shown in Fig. 2(D), were at K = 2 (0.53658). The western cluster is the area around the Kofu and Fujinomiya as the eastern end. On the other hand, the western end of the eastern cluster is around Kyoto. In the Tokai region, located between the Kanto and Kyoto regions, it was found that genetic populations were mixed on a gradient.

### Demographic history and divergence time

**Fig. 3.**
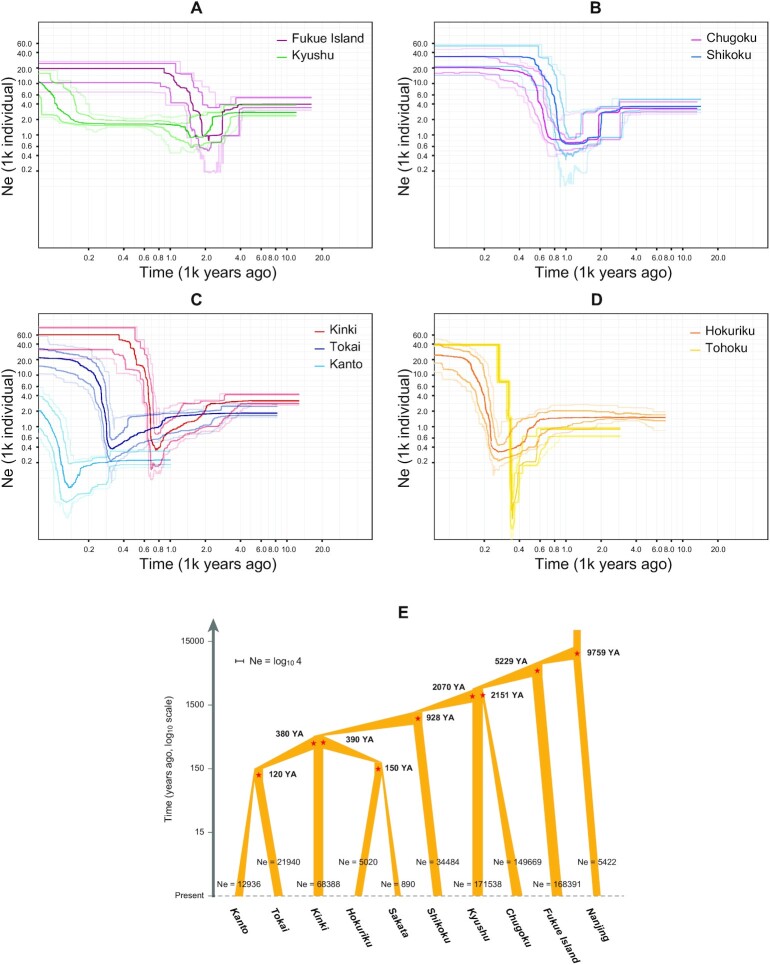
The simulated scenario of the demographic history and divergence time of *G. japonicus*. (A)–(D) Demographic history of each regional population. The horizontal axis is the reverse-direction age (1,000 years ago) on the log10 scale. The vertical axis is the number of effective populations (Ne) on the log10 scale. The most apparent lines represent the median population size for the region, and the slightly transparent lines of the same color represent 75% and 95% CI. (E) The adopted scenario, M1. The vertical axis is the reverse direction age (years ago, YA) on the log10 scale. The thickness of each population is the number of effective populations on the log10 scale. The divergence time next to the node, with the star mark and the Ne of each population, represents the median of the estimated range.

The demographic histories of the populations from each region are shown in Fig. 3. All regional populations tended to recover effective population sizes after experiencing significant bottlenecks, as shown below—Fukue Island: 3,000 years ago (Fig. 3A), Kyushu: 2,400 years ago (Fig. 3A), Chugoku: 2,000 years ago (Fig. 3B), Shikoku: 2,000 years ago (Fig. 3B), Kinki: 2,000 to 800 years ago (Fig. 3C), Tokai: 900 to 300 years ago (Fig. 3C), Kanto: 220 to 100 years ago (Fig. 3C), Hokuriku: 600 to 500 years ago (Fig. 3D), and Tohoku: 600 to 400 years ago (Fig. 3D).

The observed summary statistics are listed in Table S2, and the posterior distributions for each parameter simulated with ABCtoolbox (28) are in Table S4. A total of two scenarios (M1 and M2) were simulated with ABCtoolbox, and the adopted scenario (M1) is shown in Fig. 3(E). The rejected one (M2) is shown in Figure S2. The most probable model selected with the multinomial logistic regression (mnlogistic) method was M1 (probability = 0.83). Model M2 (probability = 0.17), in which migration was considered, was rejected. Therefore, migration between regions was considered negligible in subsequent discussions. In model M1, the median age at which each regional group diverged from its ancestral group was as follows: as Nanjing–Fukue Island, 6,506 generations; Fukue Island–Kyushu, 3,486 generations; Kyushu–Chugoku, 1,434 generations; Kyushu–Shikoku, 1,380 generations; Shikoku–Kinki, 618.5 generations; Kinki–Hokuriku, 260 generations; Kinki–Tokai, 253 generations; Hokuriku–Tohoku, 100 generations; and Tokai–Kanto, 80 generations.

## Discussion

### Genetic structure in the Japanese archipelago

Previous studies based on mitochondrial DNA and microsatellite analyses have suggested that the Japanese populations of *G. japonicus* have low genetic diversity and poor geographic structure (10). On the other hand, China has more genetic diversity than Japan, even if limited to the east coast. This was explained by the fact that *G. japonicus* is native to China and exotic to Japan, and gene flow between populations may have been facilitated by the modern development of globalized transportation networks. Since *G. japonicus* is widely distributed in eastern China, genetic diversity within China may be quite high, but this is not clear at this time. Although it is a matter of concern that only one site in China is used in this study, it is highly likely that the population on the east coast of China, which is geographically closest to the Japanese archipelago, is a lineage close to the ancestral population.

On the other hand, our high-resolution analysis using genome-wide SNPs showed geographically differentiated genetic structures of *G. japonicus* in the Japanese archipelago. In this study, almost all habitats within the Japanese archipelago were covered, and the reliability of the results for the Japanese archipelago is considered to be high. So, the discrepancy with the results of the previous study is simply due to the resolution of the loci analyzed. As distribution tends to be restricted by temperature, some geckos are geographically isolated by barriers, such as mountain ranges, and genetically differentiated even between relatively close land areas (29, 30). In the area of the Japanese archipelago considered here, most of the distribution of *G. japonicus* is in urban areas along the coast. The distribution of the species and its habitat in mountainous regions has not been confirmed (31). Seas and mountains are lined up at the boundaries of each regional group, as shown in Fig. 2(C) and (D). Additionally, as the rejected model M2 of divergence time (Fig. 3F) shows, the degree of gene flow between each was minimal. Therefore, the pattern is similar to the precedents of other gecko species. The divergence of each regional population occurred hundreds to thousands of years ago, suggesting that the influence of gene flow due to the current transportation networks on the expansion of distribution may not be significant. In other words, although genetic exchanges among each regional population have been promoted by modern transport networks, their impact is thought to be minor at the genome-wide level. It is believed that these gene structures were formed by the founder effect, originating from individuals who accidentally human-mediately broke through geographical barriers and were introduced to each region.

### Age of migration to the Japanese archipelago and dispersal history

**Fig. 4.**
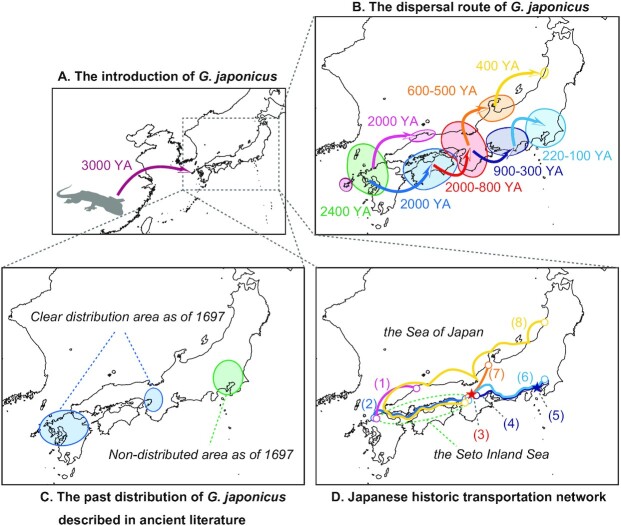
The mutual history of *G. japonicus* and humans. (A) The introduction age of *G. japonicus* from China. (B) The dispersal history of *G. japonicus* was estimated based on the timing at which each regional group experienced a bottleneck. (C) The past distribution of *G. japonicus* described in ancient literature. (D) An overview of the Japanese historic transportation network. (1) and (2) The ancient sea routes of about 2,000 years ago. (3) Heiankyo, an ancient capital established in 794 AD. (4) Tokaido, the old arterial road that flourished in the Middle Ages. (5) Kamakura Shogunate, the military government established in 1185 AD. (6) Tokaido Main Line, a railway that opened in 1889 AD. (7) A part of the national transportation network created with the development of the monetary economy in the late Middle Ages. (8) Nishimawari–Kaiun, a merchant ship route that orbits the Sea of Japan and was established in 1672 AD.

The approximate ages of the bottlenecks of each population were roughly consistent with their divergence times. The demographic history of *Aedes albopictus*, which is a mosquito that is artificially expanding around the world, is similar to that found in this study. It is known that the individuals of this species experienced a steep bottleneck when they invaded each region (32). Therefore, considering the bottleneck timing as the age of the introduction, the dispersal routes of *G. japonicus* in the Japanese archipelago are estimated with the divergence scenario (Fig. 4A and B). It is worth noting that the Tohoku population has experienced two steep bottlenecks in the past. Still, it is thought that the former event reflects the intrusion event into Hokuriku because the individuals from this region are phylogenetically closest to those from the Hokuriku population. Therefore, it is reasonable to think that the actual timing of introduction into Tohoku was the latter. In addition, despite the fact that populations derived from Kyushu in various parts of Japan must have experienced multiple bottlenecks, the dynamics before major bottlenecks were flat in all populations. This can be explained as follows. This estimation was performed based on the genetic information possessed by the current individual. However, much of the genetic information possessed by the ancestral population should have been lost in the most recent major bottleneck. Therefore, it is likely that the dynamics prior to the major bottleneck cannot be plotted in detail. However, the decreasing trend in the starting size of each regional population as one moves to a newer region suggests that the existence of multiple bottlenecks is present.

Based on Japanese human history, we verified whether the human-mediated introduction of *G. japonicus* in the above scenario was possible. An overview of major Japanese transportation networks from ancient to modern times is shown in Fig. 4(D). The estimated times at which part of the source population in China was introduced to Fukue Island and Kyushu are reasonable. It was suggested that *G. japonicus* became viable in Kyushu about 8,000 years ago (10). The Goto Islands, including Fukue Island, are a volcanic archipelago, and the last eruption of the volcano on Fukue Island occurred between 2,400 and 2,300 years ago (33). From the results of the demographic history, a pattern was observed in which the population of *G. japonicus* on Fukue Island immediately returned to its original state after a slight decrease in its effective population size during the approximate time of the eruption. This event, considering this dynamic as a temporary effect of the eruption, is a calibration point that strengthens the validity of the calculation method of the introduction age into each region. Located on the west side of Kyushu, the Goto Islands were gateways to biological exchanges with the continent. It may be that the geckos first invaded the Goto Islands a little earlier than they crossed over to the mainland of Kyushu. This era corresponds to the Jomon period (16,000 to 3,000 years ago) and the Yayoi period (3,000 to 1,700 years ago) in the classification of Japanese human history. Recent archaeological and genetic studies have suggested that rice cultivation had been propagated to Japan from Southeast Asia via China by at least the end of the Jomon period (about 3,000 years ago) (34). It is suggested that there were human exchanges between the Japanese archipelago and the continent during this period, and *G. japonicus* probably migrated to Kyushu during this time.

The introduction age to the Chugoku region and the Shikoku region corresponds to the middle to late Yayoi period in Japanese human history. Ancient Izumo in the Chugoku region prospered as a major force in Japan at that time, and many archaeological sites have been excavated in the area. Furthermore, ancient Izumo, as the Izumo mythology, appears in the oldest ancient Japanese documents, such as “Kojiki (35)” and “Nihon Shoki (36).” It is said that close exchanges with northern Kyushu through maritime traffic across the Sea of Japan were also related to social development in this region (37–39). Additionally, maritime traffic across the Seto Inland Sea (the inland sea surrounded by the Kyushu, Shikoku, Chugoku, and Kinki regions) from Kyushu to Kinki has developed since ancient times (40). *Gekko japonicus* was able to disperse from Kyushu via multiple routes by taking advantage of the ancient development of local communities and cultural exchanges.

This species took a long time to achieve an introduction to the Kinki region, but what kind of insights on that introduction can be obtained from human history? It is said that there was a blank period with almost no records 200 to 400 AD. Still, trade and transportation continued through the Asuka (592 to 710 AD), Nara (710 to 794 AD), and Heian periods (794 to 1185 AD). These were all periods when the capital was located in the Kinki region. It is reasonable to assume, considering the divergence time estimation scenario, that *G. japonicus* was spread from Shikoku along with the aforementioned maritime traffic in the Seto Inland Sea and the development of human society.

*Gekko japonicus* seems to have further expanded into the Tokai region after the introduction to the Kinki region had been mostly achieved. A major arterial road called Tokaido was fully developed during this period (41). This was a land route that ran along the coast of the Tokai region and connected the Kinki and Kanto regions. At the end of 1100s AD, the Shogunate (military government) was established in Kamakura, within the Kanto region. This era was the Kamakura period (1185 to 1333 AD). At that time, the Tokaido road functioned as an artery connecting the Shogunate with the capital of the Kinki region. From a topographical point of view, there are few geographical barriers, such as large mountains between the Shikoku, Kinki, and Tokai regions. Consequently, slow natural dispersal was active in addition to human-mediated rapid dispersal. Continuous long-term dispersal events could have created a gradual bottleneck in these regions. By 300 years ago, the geckos had completed their expansion into the Tokai region. Still, individuals of the species seem to have been stopped by the mountains that separate the Tokai and Kanto regions (including Mount Fuji, the highest peak in Japan). It was not until at least 220 years ago, at the end of the Edo period (1603 to 1868 AD), that the gecko was introduced to the Kanto region. This timeline is consistent with the description in “Honcho Shokkan,” in which geckos did not inhabit the Kanto region as of 1697 AD. Then, how did they make their way into the Kanto region? Looking at demographic dynamics, the slope of the bottleneck has become steeper since about 150 years ago, during the Meiji period (1868 to 1912 AD). The railway network began to be developed in Japan in the latter half of the 1800s AD. In 1889, the Tokaido Main Line was fully opened, operating a route similar to the Tokaido. Individuals of *G. japonicus* may have gained the ability to break through the geographical barrier presented by the high mountains by riding this modern pipeline connecting the Tokai and Kanto regions.

The age when individuals of *G. japonicus* were introduced to the Hokuriku region from the Kinki region by a route different from their spread to the Tokai and Kanto regions was the so-called “Warring States Period” (1467 to 1590 AD) in Japanese history. During that time, wars broke out all over the country. However, this period was also when the movement and distribution of people became active, and the monetary economy developed (42). Possibly, individuals of *G. japonicus* dispersed in the Hokuriku region with the development of the logistics network between the Middle Ages and the early modern period. Subsequently, these individuals reached Sakata in the Tohoku region. A sea route called “Nishimawari–Kaiun” was established in 1672 AD—merchant ships sailed westward along the coast, stopping by port towns from the Kanto to the Tohoku regions. Sakata, one of the port towns that prospered due to the development of this route (43), functioned as a key route point in the advancement of *G. japonicus* from the Hokuriku region.

## Conclusion

*Gekko japonicus* migrated from China to Kyushu before prehistoric times, spreading from west to east and south to north in the Japanese archipelago for thousands of years. This becomes clear through an approach in which human history and the natural sciences are integrated. There were many similarities between the spread of *G. japonicus* and the historical development of Japanese society, suggesting that ancient human–organism interactions are an essential factor in understanding the present distribution of these organisms. The past impact on ecosystems by humans, which has often been overlooked, is more significant than previously imagined. It is hoped that organisms worldwide will be examined from various perspectives to understand the mutual history of humans and nature.

## Materials and Methods

### Scrutiny of ancient literature

Using the digital service of the National Diet Library of Japan (https://dl.ndl.go.jp/), in which many originals and manuscripts of ancient Japanese literature are archived, we have scrutinized dictionaries and academic books from various ages, including the most ancient. We comprehensively collected the descriptions of reptiles and amphibians available from these sources.

### Sampling

From 2018 to 2021, 182 individuals of *G. japonicus* were collected from 37 sites in Japan and one in China (Figure S1). A total of three other species of the genus, *Gekko* sp. (undescribed species called Nishiyamori in Japanese), *G. tawaensis*, and *G. shibatai*, were collected from three other sites in Japan as outgroups. The individual numbers and locations of the samples used in the analysis are summarized in Table S1. To avoid destructive sampling, only autotomized tail tissue was stored in 100% ethanol, and the individuals were released on the spot. It is worth remembering that the tail of the gecko regenerates over time.

### Laboratory protocol

Total DNA was extracted from tissue pieces of the gecko tails collected according to the protocol using Nucleospin tissue (TaKaRa, Shiga Pref., Japan). The DNA was further subjected to RNase treatment. Then, double digest restricted-site associated DNA sequencing (ddRAD-seq) was conducted following Peterson et al. (2012) (44). The library was prepared with 40 ng/μL of gDNA for each sample. Samples were digested with EcoRI, MspI, P1, and P2 adapters, and each fragment was ligated. We then pooled them at equimolar concentrations and purified them using the Nucleospin gDNA clean-up kit (TaKaRa). We then selected 300 to 500 base pair (bp) fragments using Pippin Prep (Saga Science, MA, USA). The size-selected DNA fragments were amplified in polymerase chain reactions (PCR) for eight cycles using Phusion PCR reagents (New England Biolabs, MA, USA). Later, we cleaned up the reaction products and removed the PCR primers using the Nucleospin gDNA clean-up kit and Pippin Prep. The tuned ddRAD library was sequenced (150 bp paired-end) using Illumina HiSeqⅩ (Illumina, CA, USA) paired-end sequencing at Macrogen, Japan.

### De-novo assembly

Demultiplex was performed using ipyrad 0.9.14 (45) from the sequence, read and divided into individual reads. At this time, a barcode sequence mismatch was not allowed. Then, a section from the 3’ end to 5 bp of each lead was trimmed as a quality control if the Qscore was lower than 33. The clustering threshold was set to 85%, the read was clustered for each individual, and a consensus sequence was created. The minimum number of depths allowed was six, and the maximum was 10,000. The ratio of an ambiguous site (N) to a heterozygous base arranged in the consensus sequence was 0.05. The number of raw reads obtained for each individual was unexpectedly large. Therefore, only one of the paired ends (R1) was used as a single end. From the consensus sequence created for each individual, clustering was performed with a threshold of 90% to generate a consensus sequence for the entire population. Finally, a sequence with 180/185 shared loci, a bp number by indel lower than five, and a maximum SNP number per locus lower than 0.2 were extracted from this sequence, and an analysis data set (Set.Phylogeny) was created. In addition, the following datasets were prepared for the other analyses. Set.Structure1 was made from only Japanese individuals, excluding three Chinese and three outgroup individuals with 176/182 shared loci. Set.Structure2 was created from individuals from the Shikoku, Kinki, Tokai, and Kanto regions of Japan, with 73/74 shared loci. Set.Divergence was created by excluding individuals that did not form a monophyletic group in the Kyushu region from Set.Phylogeny with 166/169 shared loci.

### Phylogenetic analysis and population structure

A molecular phylogenetic tree based on the maximum likelihood method was created using iqtree 1.6.8 (46) from the loci data of Set.Phylogeny. Model selection was performed based on BIC using Model Finder Plus (47), and the Ultrafast Bootstrap was set to 2,000. We also calculated the posterior probabilities of each node based on the approximate Bayesian method. The genetic structure of the Japanese population was analyzed using ADMIXTURE (27). The data of Set.Structure1 was formatted by PLINK (48) and was used for the analysis. The number of clusters (K) was set to 1 to 30. The data of the Set.Structure2 dataset was used to clarify the more detailed population genetic structure in the Shikoku, Kinki, Tokai, and Kanto regions that formed one cluster in the higher-order structure. Then, the data were analyzed similarly using ADMIXTURE (the number of K was set to 1 to 15).

### Demographic history and divergence time

The demographic history of each regional population was estimated using Stairway plot v2 (49). Stairway plot is a population dynamics estimation software similar to Extended Bayesian Skyline Plot (50). However, it is performed based on the site frequency spectrum (SFS) and is known to provide a more accurate estimation of recent past dynamics than can be made compared to with the latter. First, the observed SFS values for each regional population were projected from the data of Set.Strucuture1 using easySFS (51). Individuals were divided into the following 11 groups based on the phylogenetic and population structure analysis results and geographical divisions (partially different from administrative divisions), such as mountains and the sea. The considered regions were (1) Tohoku: Sakata (*N* = 15); (2) Kanto: Utsunomiya, Noda, Saitama, Tama, and Kawasaki (*N* = 15); (3) Tokai: Kofu, Kai, Atami, Fujinomiya, Shizuoka, Hamamatsu, and Nagoya (*N* = 31); (4) Hokuriku: Itoigawa, Takaoka, Kanazawa, Wajima, and Suzu (*N* = 39); (5) Kinki: Uji, Kyoto, Sayo, and Sakai (*N* = 16); (6) Chugoku: Izumo (*N* = 16), (7) Shikoku: Takamatsu, Mitoyo, Kochi, and Muroto (*N* = 12); (8) Kyushu-T: Nagasaki (only one individual, s158), Fukuoka, Tsushima, Uku, and Iki (*N* = 16); (9) Fukue Island (*N* = 6); (10) Kyushu-F: other individuals of Kyushu 1 and Kyushu 2 (*N* = 13); and (11) China: Nanjing (*N* = 3).

A preliminary analysis using all individuals in Kyushu as a single population showed almost unchanged population dynamics. Thus, no bottleneck could be detected in Kyushu, even though other regional populations showed results similar to the true analyses shown in Fig. 3(B)–(D). This is the reason why paraphyletic individuals in Kyushu 1 and Kyushu 2 clades with low numbers were excluded, and two monophyletic groups with enough individuals (Kyushu-T and Fukue Island, Fig. 2A) from the Kyushu 1 clade were considered as two regional populations for the true analysis. The number of projections was set to 2N-1. Among the parameters in the input file of Stairway plot, SFS (projected value), nseq (2 N), and nrand were set at unique values for each regional population. Nrand was specified as four ranges (nseq-2)/4, (nseq-2)/2, 3*(nseq-2)/4, and (nseq-2), as recommended in the manual of Stairway plot software. All other parameters were set to the same values for all regional populations. Following a previous study (29), the mutation rate values were based on those of *Cnemaspis* geckos (0.025% Myr-1). The exact generation time of *G. japonicus* is not known, but based on data from a closely related species, *G. hokouensis*, it is known that *G. japonicus* individuals take 1 to 2 years to become sexually mature (52). Based on the above, the mutation rate for *G. japonicus* was converted to 1.5 years per generation (3.75e-8 per site per generation). All other parameters were left as defaults. Analyses were not performed for Kyushu-F, which was removed, or for China, which did not have sufficient populations.

Furthermore, divergence time estimation, based on coalescent theory, was performed from the SNP data of the Set.Divergence dataset using ABCtoolbox (28). Fastsimcoal2 (fsc26) (53) was used for the coalescent simulation, and arlsumstat (54) was used to calculate the number of statistical genetic summaries for the simulated sequence. *Fst* and *ϕ* were used as the summary statistics. For the observed data, summary statistics were calculated using arlsumstat (54) from the SNP data of the Set.Divergence dataset. Since the observed data were SNPs, minor allele frequency (*MAF*) was used as the parameter. As for the other parameters, the number of effective populations and the number of branched generations of each population were used. For each parameter used in the simulation, a noninformation prior distribution was set, and random numbers were generated from the initial distribution for each number of simulations to perform the simulation (Table S3). In each scenario, 100,000 simulations were performed. Models were selected using the mnlogistic method. The model with the highest probability was adopted. Additionally, the posterior distribution of the parameters was estimated using the rejection method in the adopted scenario. The tolerance rate was set to 0.01 in both cases. The analysis was conducted using the “abc” package (55) in R (56).

## Supplementary Material

pgac245_Supplemental_File

## Data Availability

The raw sequence data were deposited in the DDBJ Sequence Read Archive (https://www.ddbj.nig.ac.jp/dra/index.html; Submission ID: DRA014433).

## References

[1] Bullock JM et al. 2018. Human-mediated dispersal and the rewiring of spatial networks. Trends Ecol Evol. 33:958–970. 30314915 10.1016/j.tree.2018.09.008

[2] Wilson JRU , Dormontt EE, Prentis PJ, Lowe AJ, Richardson DM 2009. Something in the way you move: dispersal pathways affect invasion success. Trends Ecol Evol. 24:136–144. 19178981 10.1016/j.tree.2008.10.007

[3] Gippet JM , Liebhold AM, Fenn-Moltu G, Bertelsmeier C 2019. Human-mediated dispersal in insects. Curr Opin Insect Sci. 35:96–102. 31479895 10.1016/j.cois.2019.07.005

[4] Rödder D , Solé M, Böhme W, Forschungsmuseum Z, Adenauerallee AK 2008. Predicting the potential distributions of two alien invasive Housegeckos (Gekkonidae: *Hemidactylus frenatus, Hemidactylus mabouia*). North West J Zool. 4: 236–246.

[5] Case TJ et al. 1994. Invasions and competitive displacement among house geckos in the Tropical Pacific published by: Ecological Society of America Stable. Ecology. 75:464–477.

[6] Yeakel JD et al. 2014. Collapse of an ecological network in Ancient Egypt. Proc Natl Acad Sci USA. 111:14475–14477. 10.1073/pnas.1408471111 PMC4210013 25201967

[7] Carlton JT 1989. Man's role in changing the face of the ocean: biological invasions and implications for conservation of near-shore environments. Conserv Biol. 3:265–273.

[8] Richardson DM et al. 2011. Human-mediated introductions of Australian acacias - a global experiment in biogeography. Divers Distrib. 17:771–787.

[9] Kim DI et al. 2019. Patterns of morphological variation in the Schlegel's Japanese gecko (*Gekko japonicus*) across populations in China, Japan, and Korea. J Ecol Environ. 43:1–9.

[10] Kim JS et al. 2020. Genetic diversity and inferred dispersal history of the Schlegel's Japanese Gecko (*Gekko japonicus*) in Northeast Asia based on population genetic analyses and paleo-species distribution modelling. Mitochond DNA Part A DNA Map Seq Anal. 31:120–130. 10.1080/24701394.2020.1742332 32212876

[11] Kim DI et al. 2018. Comparisons of microhabitat use of Schlegel's Japanese gecko (*Gekko japonicus*) among three populations and four land cover types. J Ecol Environ. 42: 24.

[12] Hayashi R 2014. Past biodiversity: historical Japanese illustrations document the distribution of whales and their epibiotic barnacles. Ecol Indic. 45:687–691.

[13] Kawakatsu Y et al. 2021. Combination of genetic analysis and ancient literature survey reveals the divergence of traditional *Brassica rapa* varieties from Kyoto, Japan. Hortic Res. 8:132. 34059655 10.1038/s41438-021-00569-0 PMC8167115

[14] Arai H , 1717; Touga. https://dl.ndl.go.jp/info:ndljp/pid/993111. [last accessed January 10, 2021]

[15] Kariya E , 1827; Shakuchu Wamyo Ruiju Sho. https://dl.ndl.go.jp/info:ndljp/pid/991791. [last accessed January 10, 2021].

[16] Fukae S , 918; Honzo Wamyo. https://dl.ndl.go.jp/info:ndljp/pid/2555537/1. [last accessed January 10, 2021].

[17] Minamoto S , 931; Wamyo Ruiju Sho. https://dl.ndl.go.jp/info:ndljp/pid/2544225/1. [last accessed January 10, 2021].

[18] Sugawara K . 1081; Ruiju Myogi Sho. https://dl.ndl.go.jp/info:ndljp/pid/2586900/1. [last accessed January 10, 2021].

[19] Tachibana T . 1181; Iroha Jirui Sho. https://dl.ndl.go.jp/info:ndljp/pid/1186797/1. [last accessed January 10, 2021].

[20] Unknown, *Setuyo Shu* (1400). https://dl.ndl.go.jp/info:ndljp/pid/2532232/1. [last accessed January 10, 2021].

[21] Unknown, *Wagoku Hen* (1400).https://dl.ndl.go.jp/info:ndljp/pid/2543700/1. [last accessed January 10, 2021].

[22] Unknown, *Nippo Jisho dicrionary (Vocabulario da Lingoa de Iapam com a declara çã o em Portugues)*. 1603;

[23] Nakamura T . 1666; Kinmo Zui. https://dl.ndl.go.jp/info:ndljp/pid/2569350/1. [last accessed January 10, 2021].

[24] Ono R . 1803; Honzo Komoku Keimo. https://dl.ndl.go.jp/info:ndljp/pid/2569382/1. [last accessed January 10, 2021].

[25] Hirano H . 1697; Honcho Shokkan. https://dl.ndl.go.jp/info:ndljp/pid/2569424/1. [last accessed January 10, 2021].

[26] Minh BQ , Nguyen MAT, Von Haeseler A 2013. Ultrafast approximation for phylogenetic bootstrap. Mol Biol Evol. 30:1188–1195. 23418397 10.1093/molbev/mst024 PMC3670741

[27] Alexander DH , Lange K 2011. Enhancements to the ADMIXTURE algorithm for individual ancestry estimation. BMC Bioinf. 12: 246. 10.1186/1471-2105-12-246 PMC3146885 21682921

[28] Wegmann D , Leuenberger C, Neuenschwander S, Excoffier L 2010. ABCtoolbox: a versatile toolkit for approximate Bayesian computations. BMC Bioinf. 11: 116. 10.1186/1471-2105-11-116 PMC2848233 20202215

[29] Nguyen HN et al. 2019. Historical demography of four gecko species specializing in boulder cave habitat: implications in the evolutionary dead end hypothesis and conservation. Mol Ecol. 28: mec.14985. 10.1111/mec.14985 30580492

[30] Yan J , Wang Q, Chang Q, Ji X, Zhou K 2010. The divergence of two independent lineages of an endemic Chinese gecko, Gekko swinhonis, launched by the Qinling orogenic belt. Mol Ecol. 19:2490–2500. 20636892 10.1111/j.1365-294X.2010.04660.x

[31] Kim DI et al. 2020. Prediction of present and future distribution of the Schlegel's Japanese gecko (*Gekko japonicus*) using MaxEnt modeling. J Ecol Environ. 44:5.

[32] Sherpa S et al. 2019. Unravelling the invasion history of the Asian tiger mosquito in Europe. Mol Ecol. 28:2360–2377. 30849200 10.1111/mec.15071

[33] Nagaoka S , Furuyama K 2004. Eruptive history of the Onidake Volcano Group on Fukue Island, Western Japan. J Geogr (Chigaku Zasshi). 113:349–382.

[34] Shomura A et al. 2008. Deletion in a gene associated with grain size increased yields during rice domestication. Nat Genet. 40:1023–1028. 18604208 10.1038/ng.169

[35] Ohno Y , 712; Kojiki.

[36] Toneri P , Kodera K., 720; Nihon Shoki.

[37] Mizuno Y . 1975. The ancient Izumo and Yamato. Owa I., editor. Tokyo: Daiwa Shobo. p. 11–15.

[38] Mori K et al. 1991. The sea of Japan and the world of Izumo. Aiga T., editor. Tokyo: Shogakukan. p. 126–128.

[39] Matsuo H et al. 2005. The history of Shimane prefecture, Matsuzawa S., editor. Tokyo: Yamakawa Shuppansha. p. 34–41.

[40] Obayashi T et al. 1991. Ama culture in Setouchi. Aiga T., editor. Tokyo: Shogakukan. p. 9–18.

[41] Honda T . 2014. The Tokaido in early modern, Maeda H., editor. Tokyo: Seibundo Publishing. p. 5–30.

[42] Miyahara T et al. 2012. History of Japanese society, Council of History Educators, editor. Tokyo: Otsuki Shoten. p. 145–153.

[43] Izuta C . 1979. Yamagata prefecture, Hisaki N., editor. Tokyo: Shoheisha. p. 71–77.

[44] Peterson BK , Weber JN, Kay EH, Fisher HS, Hoekstra HE 2012. Double digest RADseq: an inexpensive method for de novo SNP discovery and genotyping in model and non-model species. PLoS ONE. 7:e37135. 22675423 10.1371/journal.pone.0037135 PMC3365034

[45] Eaton DAR , Overcast I 2020. ipyrad: interactive assembly and analysis of RADseq datasets. Bioinformatics. 36:2592–2594. 31904816 10.1093/bioinformatics/btz966

[46] Nguyen LT , Schmidt HA, Von Haeseler A, Minh BQ 2015. IQ-TREE: a fast and effective stochastic algorithm for estimating maximum-likelihood phylogenies. Mol Biol Evol. 32:268–274. 25371430 10.1093/molbev/msu300 PMC4271533

[47] Kalyaanamoorthy S , Minh BQ, Wong TKF, Von Haeseler A, Jermiin LS 2017. ModelFinder: fast model selection for accurate phylogenetic estimates. Nat Methods. 14:587–589. 28481363 10.1038/nmeth.4285 PMC5453245

[48] Purcell S et al. 2007. PLINK: a tool set for whole-genome association and population-based linkage analyses. Am J Hum Genet. 81:559–575. 17701901 10.1086/519795 PMC1950838

[49] Liu X , Fu Y-X 2015. Exploring population size changes using SNP frequency spectra. Nat Genet. 47: 555–559. 25848749 10.1038/ng.3254 PMC4414822

[50] Heled J , Drummond AJ 2008. Bayesian inference of population size history from multiple loci. BMC Evol Biol. 8:1–15. 18947398 10.1186/1471-2148-8-289 PMC2636790

[51] GitHub - isaacovercast/easySFS: Effective selection of population size projection for construction of the site frequency spectrum. Convert VCF to dadi/fastsimcoal style SFS for demographic analysis. [accessed 2022 Mar 9]. https://github.com/isaacovercast/easySFS.

[52] Okada S , Izawa M, Ota H 2002. Growth and reproduction of *Gekko hokouensis* (Reptilia: Squamata) on Okinawajima Island of the Ryukyu Archipelago, Japan. J Herpetol. 36:473–479.

[53] Excoffier L , Dupanloup I, Huerta-Sánchez E, Sousa VC, Foll M 2013. Robust demographic inference from genomic and SNP data. PLos Genet. 9:e1003905. 24204310 10.1371/journal.pgen.1003905 PMC3812088

[54] Excoffier L , Lischer HEL 2010. Arlequin suite ver 3.5: a new series of programs to perform population genetics analyses under Linux and Windows. Mol Ecol Resour. 10:564–567. 21565059 10.1111/j.1755-0998.2010.02847.x

[55] Csilléry K , François O, Blum MGB 2012. Abc: an R package for approximate Bayesian computation (ABC). Methods Ecol Evol. 3:475–479.

[56] R Core Team . 2019. An introduction to dplR. Ind Commer Train. 10:11–18.

