# Supplementary material for: The mutual history of Schlegel’s Japanese gecko (Reptilia: Squamata: Gekkonidae) and humans inscribed in genes and ancient literature
Source: PNAS Nexus. 2022 Nov 30;1(5):pgac245. doi: 10.1093/pnasnexus/pgac245 (PMC9802249; doi:10.1093/pnasnexus/pgac245)
Supplement: pgac245_Supplemental_File [file pgac245_supplemental_file.docx]

**
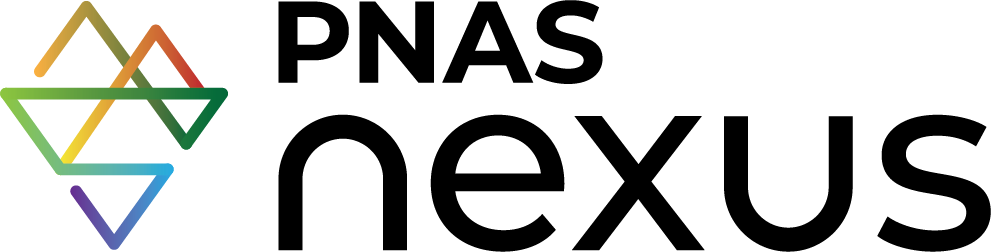
**

**Supplementary Information for**

The mutual history of Schlegel’s Japanese gecko (Reptilia: Squamata: Gekkonidae) and humans inscribed in genes and ancient literature

Minoru Chiba, Takahiro Hirano, Daishi Yamazaki, Bin Ye, Shun Ito, Osamu Kagawa, Komei Endo, Shu Nishida, Seiji Hara, Kenichiro Aratake & Satoshi Chiba

Minoru Chiba

Email: [minoru.chiba1996@gmail.com](mailto:minoru.chiba1996@gmail.com)

**This PDF file includes:**

Supplementary text

Figures S1 to S2 (not allowed for Brief Reports)

Tables S1 to S4 (not allowed for Brief Reports)

**Other supplementary materials for this manuscript include the following:**


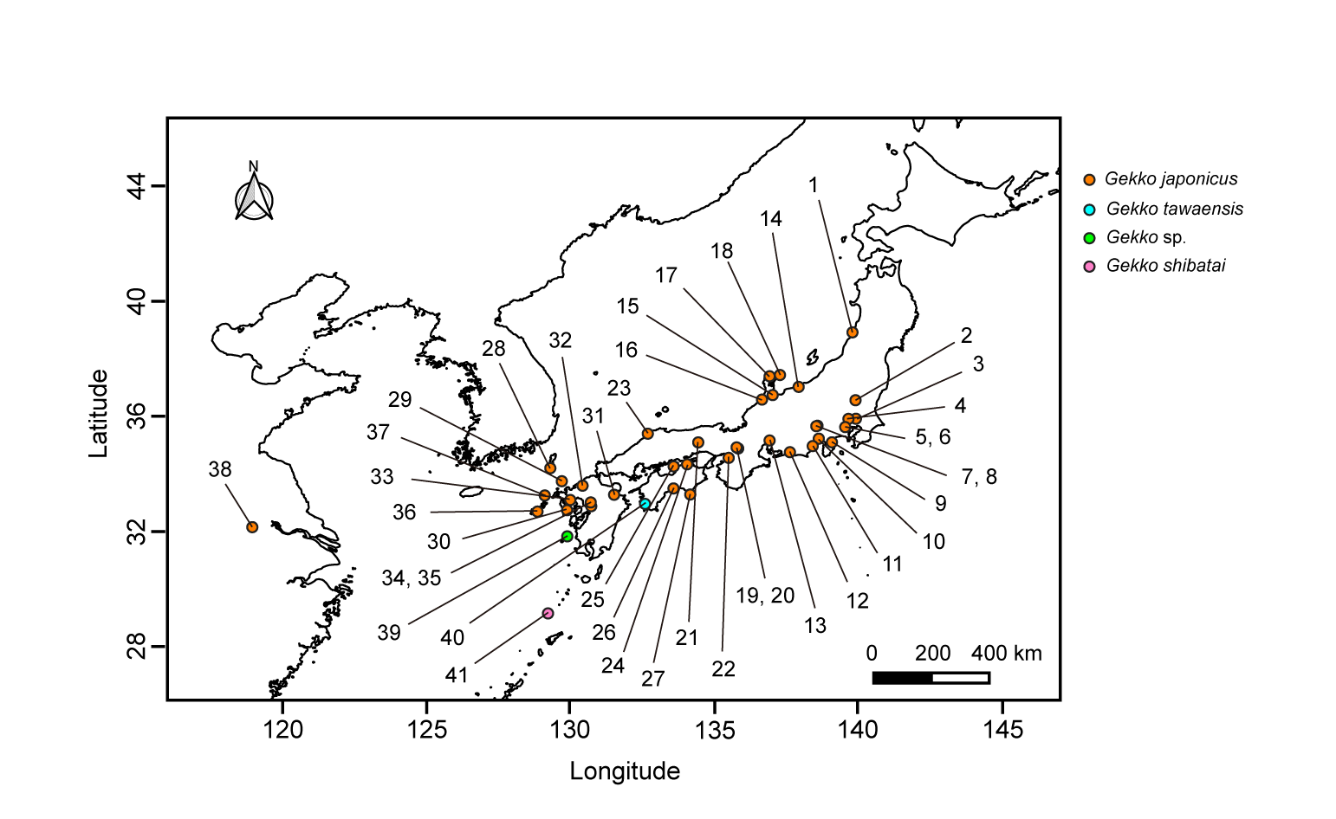


**Fig. S1.** Sampling sites of this study. The orange dots indicate where Gekko japonicus was collected. Dots of other colors indicate where the outgroup geckos were collected.

**
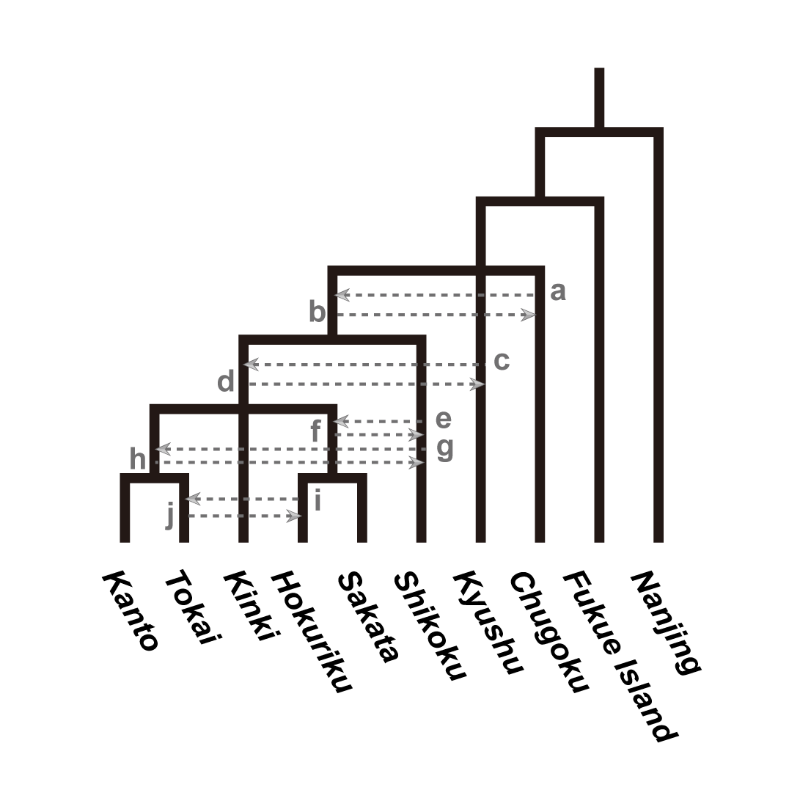
**

**Fig. S2.** The rejected scenario, M2. Migration between non-adjacent regions was considered, and they are represented by the arrows a~j.

**Table S1.** List of samples used for this study. For each sample number, the scientific name, the city where the sample was collected, the regional group (partially different from the Japanese administrative division) sorted in the analysis, the latitude and longitude are shown.

| **Sample No.** | **Accession No.** | **Species** | **Cities** | **Region** | **latitude** | **longitude** |
| --- | --- | --- | --- | --- | --- | --- |
| s1 | DRR391249 | *G. japonicus* | 1. Sakata,Yamagata | Tohoku | 38.92 | 139.831 |
| s2 | DRR391250 | *G. japonicus* | 1. Sakata,Yamagata | Tohoku | 38.92 | 139.831 |
| s3 | DRR391251 | *G. japonicus* | 1. Sakata,Yamagata | Tohoku | 38.921 | 139.831 |
| s4 | DRR391252 | *G. japonicus* | 1. Sakata,Yamagata | Tohoku | 38.911 | 139.84 |
| s5 | DRR391253 | *G. japonicus* | 1. Sakata,Yamagata | Tohoku | 38.906 | 139.841 |
| s7 | DRR391255 | *G. japonicus* | 1. Sakata,Yamagata | Tohoku | 38.905 | 139.842 |
| s8 | DRR391256 | *G. japonicus* | 1. Sakata,Yamagata | Tohoku | 38.911 | 139.837 |
| s9 | DRR391257 | *G. japonicus* | 1. Sakata,Yamagata | Tohoku | 38.913 | 139.837 |
| s10 | DRR391258 | *G. japonicus* | 1. Sakata,Yamagata | Tohoku | 38.914 | 139.84 |
| s11 | DRR391259 | *G. japonicus* | 1. Sakata,Yamagata | Tohoku | 38.914 | 139.839 |
| s12 | DRR391260 | *G. japonicus* | 1. Sakata,Yamagata | Tohoku | 38.92 | 139.842 |
| s13 | DRR391261 | *G. japonicus* | 1. Sakata,Yamagata | Tohoku | 38.92 | 139.832 |
| s14 | DRR391262 | *G. japonicus* | 1. Sakata,Yamagata | Tohoku | 38.92 | 139.831 |
| s15 | DRR391263 | *G. japonicus* | 1. Sakata,Yamagata | Tohoku | 38.919 | 139.828 |
| s16 | DRR391264 | *G. japonicus* | 1. Sakata,Yamagata | Tohoku | 38.918 | 139.829 |
| s17 | DRR391265 | *G. japonicus* | 2. Utsunomiya,Tochigi | Kanto | 36.562 | 139.886 |
| s18 | DRR391266 | *G. japonicus* | 2. Utsunomiya,Tochigi | Kanto | 36.56 | 139.895 |
| s19 | DRR391267 | *G. japonicus* | 2. Utsunomiya,Tochigi | Kanto | 36.562 | 139.883 |
| s20 | DRR391268 | *G. japonicus* | 2. Utsunomiya,Tochigi | Kanto | 36.562 | 139.885 |
| s21 | DRR391269 | *G. japonicus* | 3. Noda,Chiba | Kanto | 35.919 | 139.9 |
| s22 | DRR391270 | *G. japonicus* | 3. Noda,Chiba | Kanto | 35.919 | 139.9 |
| s23 | DRR391271 | *G. japonicus* | 3. Noda,Chiba | Kanto | 35.919 | 139.9 |
| s25 | DRR391273 | *G. japonicus* | 4. Saitama,Saitama | Kanto | 35.917 | 139.642 |
| s26 | DRR391274 | *G. japonicus* | 5. Tama,Tokyo | Kanto | 35.639 | 139.454 |
| s27 | DRR391275 | *G. japonicus* | 5. Tama,Tokyo | Kanto | 35.638 | 139.453 |
| s28 | DRR391276 | *G. japonicus* | 5. Tama,Tokyo | Kanto | 35.634 | 139.45 |
| s29 | DRR391277 | *G. japonicus* | 6. Kawasaki,Kanagawa | Kanto | 35.629 | 139.529 |
| s30 | DRR391278 | *G. japonicus* | 6. Kawasaki,Kanagawa | Kanto | 35.629 | 139.53 |
| s31 | DRR391279 | *G. japonicus* | 6. Kawasaki,Kanagawa | Kanto | 35.628 | 139.529 |
| s32 | DRR391280 | *G. japonicus* | 7. Kofu, Yamanashi | Tokai | 35.676 | 138.566 |
| s33 | DRR391281 | *G. japonicus* | 7. Kofu, Yamanashi | Tokai | 35.665 | 138.574 |
| s34 | DRR391282 | *G. japonicus* | 7. Kofu, Yamanashi | Tokai | 35.663 | 138.579 |
| s35 | DRR391283 | *G. japonicus* | 7. Kofu, Yamanashi | Tokai | 35.663 | 138.579 |
| s36 | DRR391284 | *G. japonicus* | 7. Kofu, Yamanashi | Tokai | 35.659 | 138.578 |
| s37 | DRR391285 | *G. japonicus* | 8. Kai, Yamanashi | Tokai | 35.661 | 138.525 |
| s38 | DRR391286 | *G. japonicus* | 8. Kai, Yamanashi | Tokai | 35.661 | 138.525 |
| s39 | DRR391287 | *G. japonicus* | 9. Atami,Shizuoka | Kanto | 35.1 | 139.068 |
| s40 | DRR391288 | *G. japonicus* | 10. Fujinomiya, Shizuoka | Tokai | 35.227 | 138.611 |
| s41 | DRR391289 | *G. japonicus* | 10. Fujinomiya, Shizuoka | Tokai | 35.221 | 138.607 |
| s42 | DRR391290 | *G. japonicus* | 10. Fujinomiya, Shizuoka | Tokai | 35.221 | 138.607 |
| s43 | DRR391291 | *G. japonicus* | 10. Fujinomiya, Shizuoka | Tokai | 35.223 | 138.611 |
| s44 | DRR391292 | *G. japonicus* | 11. Shizuoka, Shizuoka | Tokai | 34.97 | 138.401 |
| s45 | DRR391293 | *G. japonicus* | 11. Shizuoka, Shizuoka | Tokai | 34.981 | 138.384 |
| s46 | DRR391294 | *G. japonicus* | 11. Shizuoka, Shizuoka | Tokai | 34.978 | 138.383 |
| s47 | DRR391295 | *G. japonicus* | 11. Shizuoka, Shizuoka | Tokai | 34.978 | 138.383 |
| s48 | DRR391296 | *G. japonicus* | 11. Shizuoka, Shizuoka | Tokai | 34.978 | 138.385 |
| s49 | DRR391297 | *G. japonicus* | 12. Hamamatsu, Shizuoka | Tokai | 34.757 | 137.609 |
| s50 | DRR391298 | *G. japonicus* | 12. Hamamatsu, Shizuoka | Tokai | 34.765 | 137.614 |
| s51 | DRR391299 | *G. japonicus* | 12. Hamamatsu, Shizuoka | Tokai | 34.764 | 137.614 |
| s52 | DRR391300 | *G. japonicus* | 12. Hamamatsu, Shizuoka | Tokai | 34.763 | 137.613 |
| s53 | DRR391301 | *G. japonicus* | 12. Hamamatsu, Shizuoka | Tokai | 34.712 | 137.726 |
| s54 | DRR391302 | *G. japonicus* | 12. Hamamatsu, Shizuoka | Tokai | 34.713 | 137.726 |
| s55 | DRR391303 | *G. japonicus* | 12. Hamamatsu, Shizuoka | Tokai | 34.711 | 137.726 |
| s56 | DRR391304 | *G. japonicus* | 12. Hamamatsu, Shizuoka | Tokai | 34.713 | 137.723 |
| s57 | DRR391305 | *G. japonicus* | 12. Hamamatsu, Shizuoka | Tokai | 34.713 | 137.724 |
| s58 | DRR391306 | *G. japonicus* | 12. Hamamatsu, Shizuoka | Tokai | 34.713 | 137.724 |
| s59 | DRR391307 | *G. japonicus* | 12. Hamamatsu, Shizuoka | Tokai | 34.713 | 137.724 |
| s60 | DRR391308 | *G. japonicus* | 12. Hamamatsu, Shizuoka | Tokai | 34.713 | 137.724 |
| s61 | DRR391309 | *G. japonicus* | 13. Nagoya, Aichi | Tokai | 35.164 | 136.904 |
| s62 | DRR391310 | *G. japonicus* | 13. Nagoya, Aichi | Tokai | 35.163 | 136.903 |
| s63 | DRR391311 | *G. japonicus* | 13. Nagoya, Aichi | Tokai | 35.154 | 136.921 |
| s65 | DRR391313 | *G. japonicus* | 14. Itoigawa, Nigata | Hokuriku | 37.04 | 137.863 |
| s66 | DRR391314 | *G. japonicus* | 14. Itoigawa, Nigata | Hokuriku | 37.04 | 137.865 |
| s67 | DRR391315 | *G. japonicus* | 14. Itoigawa, Nigata | Hokuriku | 37.041 | 137.866 |
| s68 | DRR391316 | *G. japonicus* | 14. Itoigawa, Nigata | Hokuriku | 37.041 | 137.866 |
| s69 | DRR391317 | *G. japonicus* | 14. Itoigawa, Nigata | Hokuriku | 37.036 | 137.859 |
| s70 | DRR391318 | *G. japonicus* | 14. Itoigawa, Nigata | Hokuriku | 37.032 | 137.859 |
| s71 | DRR391319 | *G. japonicus* | 14. Itoigawa, Nigata | Hokuriku | 37.023 | 137.866 |
| s72 | DRR391320 | *G. japonicus* | 14. Itoigawa, Nigata | Hokuriku | 37.023 | 137.866 |
| s73 | DRR391321 | *G. japonicus* | 14. Itoigawa, Nigata | Hokuriku | 37.023 | 137.866 |
| s74 | DRR391322 | *G. japonicus* | 15. Takaoka, Toyama | Hokuriku | 36.741 | 137.01 |
| s75 | DRR391323 | *G. japonicus* | 15. Takaoka, Toyama | Hokuriku | 36.744 | 137.011 |
| s76 | DRR391324 | *G. japonicus* | 16. Kanazawa, Ishikawa | Hokuriku | 36.585 | 136.64 |
| s77 | DRR391325 | *G. japonicus* | 16. Kanazawa, Ishikawa | Hokuriku | 36.58 | 136.64 |
| s78 | DRR391326 | *G. japonicus* | 16. Kanazawa, Ishikawa | Hokuriku | 36.587 | 136.649 |
| s79 | DRR391327 | *G. japonicus* | 16. Kanazawa, Ishikawa | Hokuriku | 36.57 | 136.661 |
| s80 | DRR391328 | *G. japonicus* | 16. Kanazawa, Ishikawa | Hokuriku | 36.569 | 136.659 |
| s81 | DRR391329 | *G. japonicus* | 16. Kanazawa, Ishikawa | Hokuriku | 36.568 | 136.657 |
| s82 | DRR391330 | *G. japonicus* | 16. Kanazawa, Ishikawa | Hokuriku | 36.568 | 136.656 |
| s83 | DRR391331 | *G. japonicus* | 16. Kanazawa, Ishikawa | Hokuriku | 36.565 | 136.655 |
| s84 | DRR391332 | *G. japonicus* | 17. Wajima, Ishikawa | Hokuriku | 37.395 | 136.905 |
| s85 | DRR391333 | *G. japonicus* | 17. Wajima, Ishikawa | Hokuriku | 37.397 | 136.899 |
| s86 | DRR391334 | *G. japonicus* | 17. Wajima, Ishikawa | Hokuriku | 37.398 | 136.897 |
| s87 | DRR391335 | *G. japonicus* | 17. Wajima, Ishikawa | Hokuriku | 37.395 | 136.906 |
| s88 | DRR391336 | *G. japonicus* | 17. Wajima, Ishikawa | Hokuriku | 37.395 | 136.906 |
| s89 | DRR391337 | *G. japonicus* | 17. Wajima, Ishikawa | Hokuriku | 37.395 | 136.906 |
| s90 | DRR391338 | *G. japonicus* | 17. Wajima, Ishikawa | Hokuriku | 37.392 | 136.893 |
| s91 | DRR391339 | *G. japonicus* | 17. Wajima, Ishikawa | Hokuriku | 37.393 | 136.895 |
| s92 | DRR391340 | *G. japonicus* | 17. Wajima, Ishikawa | Hokuriku | 37.391 | 136.897 |
| s93 | DRR391341 | *G. japonicus* | 17. Wajima, Ishikawa | Hokuriku | 37.389 | 136.898 |
| s94 | DRR391342 | *G. japonicus* | 17. Wajima, Ishikawa | Hokuriku | 37.386 | 136.898 |
| s95 | DRR391343 | *G. japonicus* | 18. Suzu, Ishikawa | Hokuriku | 37.439 | 137.264 |
| s96 | DRR391344 | *G. japonicus* | 18. Suzu, Ishikawa | Hokuriku | 37.437 | 137.261 |
| s97 | DRR391345 | *G. japonicus* | 18. Suzu, Ishikawa | Hokuriku | 37.437 | 137.259 |
| s98 | DRR391346 | *G. japonicus* | 18. Suzu, Ishikawa | Hokuriku | 37.436 | 137.261 |
| s99 | DRR391347 | *G. japonicus* | 18. Suzu, Ishikawa | Hokuriku | 37.435 | 137.261 |
| s100 | DRR391348 | *G. japonicus* | 18. Suzu, Ishikawa | Hokuriku | 37.429 | 137.253 |
| s101 | DRR391349 | *G. japonicus* | 18. Suzu, Ishikawa | Hokuriku | 37.436 | 137.264 |
| s102 | DRR391350 | *G. japonicus* | 18. Suzu, Ishikawa | Hokuriku | 37.437 | 137.265 |
| s103 | DRR391351 | *G. japonicus* | 18. Suzu, Ishikawa | Hokuriku | 37.437 | 137.265 |
| s104 | DRR391352 | *G. japonicus* | 19. Uji, Kyoto | Kinki | 34.887 | 135.814 |
| s105 | DRR391353 | *G. japonicus* | 19. Uji, Kyoto | Kinki | 34.891 | 135.81 |
| s106 | DRR391354 | *G. japonicus* | 19. Uji, Kyoto | Kinki | 34.888 | 135.809 |
| s107 | DRR391355 | *G. japonicus* | 19. Uji, Kyoto | Kinki | 34.888 | 135.803 |
| s108 | DRR391356 | *G. japonicus* | 20. Kyoto, Kyoto | Kinki | 34.923 | 135.758 |
| s109 | DRR391357 | *G. japonicus* | 21. Sayo, Hyogo | Kinki | 35.098 | 134.427 |
| s110 | DRR391358 | *G. japonicus* | 21. Sayo, Hyogo | Kinki | 35.098 | 134.427 |
| s111 | DRR391359 | *G. japonicus* | 22. Sakai, Osaka | Kinki | 34.56 | 135.483 |
| s112 | DRR391360 | *G. japonicus* | 22. Sakai, Osaka | Kinki | 34.559 | 135.479 |
| s113 | DRR391361 | *G. japonicus* | 22. Sakai, Osaka | Kinki | 34.559 | 135.479 |
| s114 | DRR391362 | *G. japonicus* | 22. Sakai, Osaka | Kinki | 34.56 | 135.483 |
| s115 | DRR391363 | *G. japonicus* | 22. Sakai, Osaka | Kinki | 34.56 | 135.483 |
| s116 | DRR391364 | *G. japonicus* | 22. Sakai, Osaka | Kinki | 34.559 | 135.483 |
| s117 | DRR391365 | *G. japonicus* | 22. Sakai, Osaka | Kinki | 34.559 | 135.484 |
| s118 | DRR391366 | *G. japonicus* | 22. Sakai, Osaka | Kinki | 34.56 | 135.485 |
| s119 | DRR391367 | *G. japonicus* | 22. Sakai, Osaka | Kinki | 34.56 | 135.483 |
| s120 | DRR391368 | *G. japonicus* | 23. Izumo, Shimane | Chugoku | 35.4 | 132.676 |
| s121 | DRR391369 | *G. japonicus* | 23. Izumo, Shimane | Chugoku | 35.4 | 132.676 |
| s122 | DRR391370 | *G. japonicus* | 23. Izumo, Shimane | Chugoku | 35.398 | 132.686 |
| s123 | DRR391371 | *G. japonicus* | 23. Izumo, Shimane | Chugoku | 35.398 | 132.686 |
| s124 | DRR391372 | *G. japonicus* | 23. Izumo, Shimane | Chugoku | 35.398 | 132.686 |
| s125 | DRR391373 | *G. japonicus* | 23. Izumo, Shimane | Chugoku | 35.398 | 132.686 |
| s126 | DRR391374 | *G. japonicus* | 23. Izumo, Shimane | Chugoku | 35.398 | 132.686 |
| s127 | DRR391375 | *G. japonicus* | 23. Izumo, Shimane | Chugoku | 35.398 | 132.686 |
| s128 | DRR391376 | *G. japonicus* | 23. Izumo, Shimane | Chugoku | 35.398 | 132.686 |
| s129 | DRR391377 | *G. japonicus* | 23. Izumo, Shimane | Chugoku | 35.398 | 132.686 |
| s130 | DRR391378 | *G. japonicus* | 23. Izumo, Shimane | Chugoku | 35.393 | 132.687 |
| s131 | DRR391379 | *G. japonicus* | 23. Izumo, Shimane | Chugoku | 35.507 | 132.86 |
| s132 | DRR391380 | *G. japonicus* | 23. Izumo, Shimane | Chugoku | 35.365 | 132.751 |
| s133 | DRR391381 | *G. japonicus* | 23. Izumo, Shimane | Chugoku | 35.364 | 132.756 |
| s134 | DRR391382 | *G. japonicus* | 23. Izumo, Shimane | Chugoku | 35.371 | 132.755 |
| s135 | DRR391383 | *G. japonicus* | 23. Izumo, Shimane | Chugoku | 35.374 | 132.758 |
| s136 | DRR391384 | *G. japonicus* | 24. Takamatsu, Kagawa | Shikoku | 34.336 | 134.041 |
| s137 | DRR391385 | *G. japonicus* | 24. Takamatsu, Kagawa | Shikoku | 34.335 | 134.048 |
| s138 | DRR391386 | *G. japonicus* | 24. Takamatsu, Kagawa | Shikoku | 34.347 | 134.045 |
| s139 | DRR391387 | *G. japonicus* | 24. Takamatsu, Kagawa | Shikoku | 34.359 | 134.104 |
| s140 | DRR391388 | *G. japonicus* | 24. Takamatsu, Kagawa | Shikoku | 34.359 | 134.104 |
| s141 | DRR391389 | *G. japonicus* | 24. Takamatsu, Kagawa | Shikoku | 34.359 | 134.104 |
| s142 | DRR391390 | *G. japonicus* | 24. Takamatsu, Kagawa | Shikoku | 34.358 | 134.102 |
| s143 | DRR391391 | *G. japonicus* | 25. Mitoyo, Kagawa | Shikoku | 34.264 | 133.574 |
| s144 | DRR391392 | *G. japonicus* | 26. Kochi, Kochi | Shikoku | 33.499 | 133.568 |
| s145 | DRR391393 | *G. japonicus* | 26. Kochi, Kochi | Shikoku | 33.495 | 133.57 |
| s146 | DRR391394 | *G. japonicus* | 26. Kochi, Kochi | Shikoku | 33.494 | 133.566 |
| s147 | DRR391395 | *G. japonicus* | 27. Muroto, Kochi | Shikoku | 33.288 | 134.149 |
| s150 | DRR391398 | *G. japonicus* | 28. Tsushima, Nagasaki | Kyushu | 34.202 | 129.292 |
| s151 | DRR391399 | *G. japonicus* | 28. Tsushima, Nagasaki | Kyushu | 34.112 | 129.211 |
| s152 | DRR391400 | *G. japonicus* | 28. Tsushima, Nagasaki | Kyushu | 34.204 | 129.292 |
| s153 | DRR391401 | *G. japonicus* | 29. Iki, Nagasaki | Kyushu | 33.749 | 129.691 |
| s154 | DRR391402 | *G. japonicus* | 29. Iki, Nagasaki | Kyushu | 33.746 | 129.689 |
| s155 | DRR391403 | *G. japonicus* | 29. Iki, Nagasaki | Kyushu | 33.814 | 129.759 |
| s156 | DRR391404 | *G. japonicus* | 30. Nagasaki, Nagasaki | Kyushu | 32.742 | 129.875 |
| s157 | DRR391405 | *G. japonicus* | 30. Nagasaki, Nagasaki | Kyushu | 32.741 | 129.878 |
| s158 | DRR391406 | *G. japonicus* | 30. Nagasaki, Nagasaki | Kyushu | 32.752 | 129.881 |
| s159 | DRR391407 | *G. japonicus* | 31. Beppu, Oita | Kyushu | 33.279 | 131.507 |
| s160 | DRR391408 | *G. japonicus* | 31. Beppu, Oita | Kyushu | 33.273 | 131.506 |
| s161 | DRR391409 | *G. japonicus* | 31. Beppu, Oita | Kyushu | 33.288 | 131.495 |
| s162 | DRR391410 | *G. japonicus* | 32. Fukuoka, Fukuoka | Kyushu | 33.593 | 130.411 |
| s163 | DRR391411 | *G. japonicus* | 32. Fukuoka, Fukuoka | Kyushu | 33.59 | 130.404 |
| s164 | DRR391412 | *G. japonicus* | 32. Fukuoka, Fukuoka | Kyushu | 33.596 | 130.413 |
| s165 | DRR391413 | *G. japonicus* | 33. Ureshino, Saga | Kyushu | 33.097 | 129.986 |
| s166 | DRR391414 | *G. japonicus* | 33. Ureshino, Saga | Kyushu | 33.097 | 129.986 |
| s167 | DRR391415 | *G. japonicus* | 33. Ureshino, Saga | Kyushu | 33.097 | 129.986 |
| s168 | DRR391416 | *G. japonicus* | 33. Ureshino, Saga | Kyushu | 33.097 | 129.986 |
| s169 | DRR391417 | *G. japonicus* | 33. Ureshino, Saga | Kyushu | 33.097 | 129.986 |
| s170 | DRR391418 | *G. japonicus* | 33. Ureshino, Saga | Kyushu | 33.097 | 129.986 |
| s171 | DRR391419 | *G. japonicus* | 34. Kumamoto, Kumamoto | Kyushu | 32.881 | 130.711 |
| s172 | DRR391420 | *G. japonicus* | 35. Yamaga, Kumamoto | Kyushu | 33.012 | 130.695 |
| s173 | DRR391421 | *G. japonicus* | 36. Fukue, Nagasaki | Kyushu | 32.695 | 128.847 |
| s174 | DRR391422 | *G. japonicus* | 36. Fukue, Nagasaki | Kyushu | 32.753 | 128.761 |
| s175 | DRR391423 | *G. japonicus* | 36. Fukue, Nagasaki | Kyushu | 32.753 | 128.761 |
| s176 | DRR391424 | *G. japonicus* | 36. Fukue, Nagasaki | Kyushu | 32.694 | 128.844 |
| s177 | DRR391425 | *G. japonicus* | 36. Fukue, Nagasaki | Kyushu | 32.688 | 128.853 |
| s178 | DRR391426 | *G. japonicus* | 36. Fukue, Nagasaki | Kyushu | 32.753 | 128.761 |
| s179 | DRR391427 | *G. japonicus* | 37. Uku, Nagasaki | Kyushu | 33.254 | 129.099 |
| s181 | DRR391429 | *G. japonicus* | 37. Uku, Nagasaki | Kyushu | 33.254 | 129.099 |
| s182 | DRR391430 | *G. japonicus* | 37. Uku, Nagasaki | Kyushu | 33.264 | 129.129 |
| s183 | DRR391431 | *G. japonicus* | 37. Uku, Nagasaki | Kyushu | 33.264 | 129.129 |
| s184 | DRR391432 | *G. japonicus* | 37. Uku, Nagasaki | Kyushu | 33.265 | 129.132 |
| s185 | DRR391433 | *G. japonicus* | 37. Uku, Nagasaki | Kyushu | 33.268 | 129.118 |
| s187 | DRR391435 | *G. japonicus* | 38. Nanjing, China | China | 32.152 | 118.948 |
| s188 | DRR391436 | *G. japonicus* | 38. Nanjing, China | China | 32.152 | 118.948 |
| s189 | DRR391437 | *G. japonicus* | 38. Nanjing, China | China | 32.152 | 118.948 |
| s190 | DRR391438 | *Gekko* sp*.* | 39. Kamikoshikijima, Kagoshima | Kyushu | 31.833 | 129.882 |
| s191 | DRR391439 | *G. tawaensis* | 40. Minamiuwa, Ehime | Shikoku | 32.961 | 132.581 |
| s192 | DRR391440 | *G. shibatai* | 41. Takarajima, Kagoshima | Kyushu | 29.157 | 129.212 |

**Table S2.** Observed summary statistics. The population number is 1: Nanjing, 2: Fukue Island, 3: Kyushu-T, 4: Chugoku, 5: Shikoku, 6: Kinki, 7: Hokuriku, 8: Tokai, 9: Kanto, 10: Tohoku.

| mean_K | 0 |  | Pi_5 | 457.208 |  | FST_7_5 | 0.037961 |
| --- | --- | --- | --- | --- | --- | --- | --- |
| sd_K | 0 |  | Pi_6 | 479.385 |  | FST_7_6 | 0.112544 |
| tot_K | 0 |  | Pi_7 | 495.967 |  | FST_8_1 | 0.153944 |
| mean_H | 0 |  | Pi_8 | 472.462 |  | FST_8_2 | 0.158682 |
| sd_H | 0 |  | Pi_9 | 532.985 |  | FST_8_3 | 0.107446 |
| tot_H | 0 |  | Pi_10 | 628.467 |  | FST_8_4 | 0.082519 |
| prS_1 | 0 |  | mean_Pi | 401.126 |  | FST_8_5 | 0.098439 |
| prS_2 | 0 |  | sd_Pi | 174.238 |  | FST_8_6 | 0.070669 |
| prS_3 | 0 |  | FST_2_1 | 0.264146 |  | FST_8_7 | 0.088496 |
| prS_4 | 0 |  | FST_3_1 | 0.175749 |  | FST_9_1 | 0.171654 |
| prS_5 | 0 |  | FST_3_2 | 0.033407 |  | FST_9_2 | 0.235457 |
| prS_6 | 0 |  | FST_4_1 | 0.175199 |  | FST_9_3 | 0.161806 |
| prS_7 | 0 |  | FST_4_2 | 0.108861 |  | FST_9_4 | 0.1385 |
| prS_8 | 0 |  | FST_4_3 | 0.068237 |  | FST_9_5 | 0.143014 |
| prS_9 | 0 |  | FST_5_1 | 0.159174 |  | FST_9_6 | 0.133362 |
| prS_10 | 0 |  | FST_5_2 | 0.048591 |  | FST_9_7 | 0.126742 |
| mean_S | 0 |  | FST_5_3 | 0.018468 |  | FST_9_8 | 0.05274 |
| sd_S | 0 |  | FST_5_4 | 0.062563 |  | FST_10_1 | 0.363994 |
| tot_S | 30477 |  | FST_6_1 | 0.232325 |  | FST_10_2 | 0.362984 |
| mean_D | 0 |  | FST_6_2 | 0.189326 |  | FST_10_3 | 0.320936 |
| sd_D | 0 |  | FST_6_3 | 0.121742 |  | FST_10_4 | 0.278507 |
| mean_FS | 0 |  | FST_6_4 | 0.116349 |  | FST_10_5 | 0.299032 |
| sd_FS | 0 |  | FST_6_5 | 0.149985 |  | FST_10_6 | 0.274422 |
| Pi_1 | 149.347 |  | FST_7_1 | 0.165169 |  | FST_10_7 | 0.266529 |
| Pi_2 | 63.1218 |  | FST_7_2 | 0.076414 |  | FST_10_8 | 0.188675 |
| Pi_3 | 385.127 |  | FST_7_3 | 0.03775 |  | FST_10_9 | 0.232314 |
| Pi_4 | 347.186 |  | FST_7_4 | 0.064931 |  |  |  |

**Table S3.** Prior distribution of parameters used for divergence time estimation. All parameters were given a uniform distribution (unif) from the lower bound to the upper bound.

| **Parameters** | **Distribution** | **S1** | | **S2** | |
| --- | --- | --- | --- | --- | --- |
|  |  | **Minimum** | **Max** | **Minimum** | **Max** |
| MAF | unif | 0.001 | 0.5 | 0.001 | 0.5 |
| log10_Nanjing | unif | 2 | 6 | 2 | 6 |
| log10_Fukuejima | unif | 3 | 7 | 3 | 7 |
| log10_Kyushu | unif | 3 | 7 | 3 | 7 |
| log10_Chugoku | unif | 3 | 7 | 3 | 7 |
| log10_Shikoku | unif | 2 | 6 | 2 | 6 |
| log10_Kinki | unif | 3 | 7 | 3 | 7 |
| log10_Hokuriku | unif | 2 | 6 | 2 | 6 |
| log10_Tokai | unif | 2 | 6 | 2 | 6 |
| log10_Kanto | unif | 2 | 6 | 2 | 6 |
| log10_Tohoku | unif | 2 | 6 | 2 | 6 |
| log10_t (Tokai-Kanto) | unif | 1.5 | 3 | 1.5 | 3 |
| log10_t (Hokuriku-Tohoku) | unif | 1.5 | 3 | 1.5 | 3 |
| log10_t (Kinki-Tokai) | unif | 2 | 4 | 2 | 4 |
| log10_t (Kinki-Hokuriku) | unif | 2 | 4 | 2 | 4 |
| log10_t (Kinki-Shikoku) | unif | 2 | 4 | 2 | 4 |
| log10_t (Shikoku-Kyushu) | unif | 2 | 4 | 2 | 4 |
| log10_t (Kyushu-Chugoku) | unif | 2.3 | 4 | 2.3 | 4 |
| log10_t (Fukue-Kyushu) | unif | 3 | 4 | 3 | 4 |
| log10_t (Nanjing-Fukue) | unif | 3 | 4 | 3 | 4 |
| log10_a | unif | - | - | -15 | -11 |
| log10_b | unif | - | - | -15 | -11 |
| log10_c | unif | - | - | -15 | -11 |
| log10_d | unif | - | - | -15 | -11 |
| log10_e | unif | - | - | -15 | -11 |
| log10_f | unif | - | - | -15 | -11 |
| log10_g | unif | - | - | -15 | -11 |
| log10_h | unif | - | - | -15 | -11 |
| log10_i | unif | - | - | -15 | -11 |
| log10_j | unif | - | - | -15 | -11 |

**Table S4**. Posterior distribution for each parameter simulated by ABC toolbox. The median, standard deviation, and 95% BCI (bottom 2.5% and top 2.5%) are summarized.

| **Parameters** | **S1** | | | | **S2** | | | |
| --- | --- | --- | --- | --- | --- | --- | --- | --- |
|  | **Median** | **sd** | **0.025 BCI** | **0.975 BCI** | **Median** | **sd** | **0.025 BCI** | **0.975 BCI** |
| MAF | 0.2471965 | 0.1446179 | 0.0126980 | 0.4877831 | 0.2504165 | 0.1435474 | 0.01383063 | 0.48882433 |
| Nanjing | 5422 | 17335.11 | 129 | 61194.52 | 6608.5 | 17322.71 | 131 | 59837.8 |
| Fukuejima | 168391 | 2152355 | 2235.825 | 8103024.1 | 170364.5 | 2220956 | 2334.775 | 8438453.475 |
| Kyushu | 171538 | 2125552 | 3527.925 | 8120696.8 | 159386.5 | 2102830 | 3791.9 | 8085417.4 |
| Chugoku | 149669 | 2107162 | 1893.9 | 7960816.2 | 129445.5 | 2087710 | 1692.9 | 7972953 |
| Shikoku | 34483.5 | 230063.6 | 851.925 | 852486.05 | 35756 | 226736.6 | 1001.975 | 836263.75 |
| Kinki | 68388 | 1955754 | 1303.95 | 7622225.2 | 90661 | 1984377 | 1497.825 | 7642719.05 |
| Hokuriku | 5020.5 | 184499.6 | 227 | 748690.9 | 8213.5 | 198886.8 | 257 | 779911 |
| Tokai | 21940 | 220555.1 | 501.975 | 840032.25 | 23680 | 218656.4 | 514.975 | 824810 |
| Kanto | 12935.5 | 210098.3 | 175.975 | 800921.75 | 13372.5 | 212157.7 | 181.975 | 815196.85 |
| Tohoku | 890 | 155516.4 | 110 | 637445.3 | 1382.5 | 167506 | 113 | 675582.1 |
| t (Tokai-Kanto) | 80 | 108.6256 | 33 | 426 | 83 | 130.4955 | 33 | 528.05 |
| t (Hokuriku-Tohoku) | 100 | 131.4961 | 34 | 543.025 | 106 | 152.4584 | 34 | 629.05 |
| t (Kinki-Tokai) | 253 | 297.9262 | 105 | 1168.025 | 294 | 435.9764 | 106 | 1634.15 |
| t (Kinki-Hokuriku) | 260 | 318.4838 | 106 | 1248.3 | 308 | 437.1724 | 108 | 1660.05 |
| t (Shikoku-Kinki) | 618.5 | 605.4085 | 185 | 2457.075 | 855 | 921.2764 | 213 | 3750 |
| t (Kyushu-Shikoku) | 1380 | 1345.095 | 325 | 5463.15 | 1667.5 | 1424.605 | 384 | 5815.3 |
| t (Kyushu-Chugoku) | 1434 | 1323.038 | 338.975 | 5385.05 | 693.5 | 1138.353 | 213 | 4478.075 |
| t (Fukue-Kyushu) | 3485.5 | 1992.775 | 1134.975 | 8372.075 | 3503 | 1999.646 | 1143.975 | 8338.1 |
| t (Nanjing-Fukue) | 6505.5 | 2294.071 | 1954.95 | 9830.025 | 6430 | 2317.965 | 1918.975 | 9848.025 |
| a | - | - | - | - | 1.01E-13 | 2.06E-12 | 1.24E-15 | 7.92E-12 |
| b | - | - | - | - | 9.06E-14 | 2.07E-12 | 1.24E-15 | 8.02E-12 |
| c | - | - | - | - | 9.76E-14 | 2.08E-12 | 1.24E-15 | 8.14E-12 |
| d | - | - | - | - | 1.01E-13 | 2.03E-12 | 1.24E-15 | 7.82E-12 |
| e | - | - | - | - | 9.27E-14 | 2.02E-12 | 1.25E-15 | 7.86E-12 |
| f | - | - | - | - | 1.07E-13 | 2.09E-12 | 1.24E-15 | 8.08E-12 |
| g | - | - | - | - | 1.01E-13 | 2.04E-12 | 1.27E-15 | 7.95E-12 |
| h | - | - | - | - | 1.03E-13 | 2.09E-12 | 1.28E-15 | 8.07E-12 |
| i | - | - | - | - | 9.81E-14 | 2.03E-12 | 1.28E-15 | 7.80E-12 |
| j | - | - | - | - | 1.01E-13 | 2.08E-12 | 1.26E-15 | 7.97E-12 |
